# Draft Genome Sequence of Probiotic *Lactobacillus acidophilus* Strain L-55 Isolated from a Healthy Human Gut

**DOI:** 10.1128/genomeA.01357-16

**Published:** 2016-12-08

**Authors:** Yusuke Fujii, Hidehiro Toh, Takehiro Matsubara, Shuta Tomida, Co Thi Kim Nguyen, Iyo Mimura, Shoji Nakamura, Hidetoshi Morita

**Affiliations:** a Graduate School of Environmental and Life Science, Okayama University, Okayama, Japan; b Fundamental Laboratory, Ohayo Dairy Products Co., Ltd., Okayama, Japan; c Medical Institute of Bioregulation, Kyushu University, Fukuoka, Japan; d Okayama University Hospital Biobank, Okayama University Hospital, Okayama, Japan; e Graduate School of Medicine Dentistry and Pharmaceutical Sciences, Okayama University, Okayama, Japan; f Department of Biology, College of Education, Hue University, Hue, Vietnam

## Abstract

Probiotic *Lactobacillus acidophilus* L-55 was isolated from a healthy human gut. Here, we report the draft genome sequence of this organism.

## GENOME ANNOUNCEMENT

*Lactobacillus acidophilus*, which is a Gram-positive bacterium, is often used in dairy products as a probiotic lactic acid bacterium that benefits the health of the host (1, 2). *L. acidophilus* strain L-55, which was isolated from healthy infant feces (3, 4), has tolerance to gastrointestinal conditions and a high ability to adhere to Caco-2 cells (3, 4), and it has been used as a starter culture for commercial dairy products (3). It has also been reported that *L. acidophilus* L-55 has the effect of alleviating the symptoms of allergic rhinitis (3, 5) and atopic dermatitis (4).

The *L. acidophilus* L-55 genome was paired-end sequenced using Illumina’s MiSeq platform. Genomic libraries containing 600- to 1,000-bp inserts were constructed and sequenced, yielding 1,981,953 reads that provided 296-fold coverage of the genome. The sequence reads were assembled using the CLC Genomics Workbench version 9.0.1, and the assembled genome consists of 25 contigs with a total length of 2,009,507 bp. The genome has a G+C content of 34.6%. These features were very similar to those of 15 other sequenced *L. acidophilus* strains, except strain CFH, consistent with a previous report that *L. acidophilus* lacks genomic diversity compared to other *L. acidophilus* group species (6). The draft genome of *L. acidophilus* L-55 contained 1,884 predicted protein-coding genes, 1,760 (93%) of which were shared with the complete genome sequence of *L. acidophilus* NCFM (7). The genome information of this species will be useful for further studies of its physiology, taxonomy, and ecology.

### Accession number(s).

The draft genome sequence for *L. acidophilus* L-55 has been deposited in the DDBJ/GenBank/EMBL database under accession numbers BDHM01000001 to BDHM01000025.
